# Increased tolerance to humans among disturbed wildlife

**DOI:** 10.1038/ncomms9877

**Published:** 2015-11-16

**Authors:** Diogo S. M. Samia, Shinichi Nakagawa, Fausto Nomura, Thiago F. Rangel, Daniel T. Blumstein

**Affiliations:** 1 Department of Ecology, Federal University of Goiás, CP. 131, 74001-970 Goiânia, Brazil; 2 Department of Zoology, University of Otago, 340 Great King Street, Dunedin 9054, New Zealand; 3 Evolution and Ecology Research Centre, School of Biological, Earth and Environmental Sciences, University of New South Wales, Sydney, New South Wales 2052, Australia; 4 Department of Ecology and Evolutionary Biology, University of California, 621 Young Drive South, Los Angeles, California 90095-1606, USA

## Abstract

Human disturbance drives the decline of many species, both directly and indirectly. Nonetheless, some species do particularly well around humans. One mechanism that may explain coexistence is the degree to which a species tolerates human disturbance. Here we provide a comprehensive meta-analysis of birds, mammals and lizards to investigate species tolerance of human disturbance and explore the drivers of this tolerance in birds. We find that, overall, disturbed populations of the three major taxa are more tolerant of human disturbance than less disturbed populations. The best predictors of the direction and magnitude of bird tolerance of human disturbance are the type of disturbed area (urbanized birds are more tolerant than rural or suburban populations) and body mass (large birds are more tolerant than small birds). By identifying specific features associated with tolerance, these results guide evidence-based conservation strategies to predict and manage the impacts of increasing human disturbance on birds.

## Abstract (web summary)

Some animal species tolerate the presence of humans better than others. In a meta-analysis, Samia *et al*. find that populations of wildlife exposed to greater levels of human disturbance are more tolerant than undisturbed populations, with large birds in urbanized areas showing the highest levels of tolerance.

## Graphical abstract

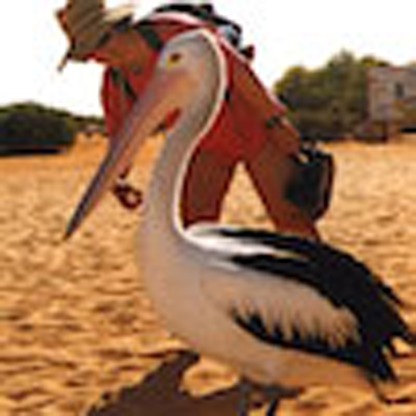

Animals often perceive humans as predators^1^. Thus, even non-consumptive human activities may affect their behaviour^2^,^3^ and generate population-level consequences^4^,^5^. For instance, declining populations of European and Australian birds are less tolerant of humans, as evidenced by them fleeing at greater distances from an approaching human (that is, flight initiation distance—FID), whereas populations from more tolerant species are increasing^6^,^7^. In addition, a synergic interaction between human tolerance and increased urbanization is expected^8^,^9^, resulting in an increased negative effect for less tolerant species. Hence, there is an urgent need to understand the factors that explain species tolerance of humans and to develop predictive models of tolerance^10^. One strategy to do so is to identify traits associated with species tolerance (that is, reduced responsiveness to people in areas with more people) or intolerance (that is, increased responsiveness to people in areas with more people) to human presence, which can be done by capitalizing on studies that compare the responses of animals in areas with varying levels of human presence.

We conducted a meta-analysis of 75 studies on birds (180 species), mammals (16 species) and lizards (16 species) to comprehensively evaluate species' tolerance of human disturbance. To address the potential non-independence among effect sizes, we incorporated study identity and species phylogeny as random factors. For each observation, we calculated the Hedges' g^11^, a measure of effect size, by comparing the mean FID of a population observed in areas of higher human disturbance with the mean FID of a population observed in areas of lower human disturbance. Negative effect sizes indicate tolerance of human disturbance, whereas positive effect sizes indicate intolerance of human disturbance. For the best-studied taxa—birds—we used a multi-model inference approach^12^ to identify the most important predictors of tolerance of human disturbance. Specifically, we tested for the effects of body mass, clutch size, habitat openness, group size, foraging habit, diet, migration behaviour and the type of habitats contrasted in populations under low and high human disturbance (for example, rural versus urban, natural versus urban; hereafter referred to as ‘habitat contrast') on bird's tolerance of human disturbance. Because it is well known that urbanization is a correlate of avian extinction^13^, we also conducted a separate meta-analysis focusing on bird populations from the rural–urban habitat contrast (the best studied contrast) to investigate if traits were similarly likely to explain differential behavioural responses^13^,^14^.

Here we find that, overall, disturbed populations of animals of the three major taxa are more tolerant of human disturbance than undisturbed (or less disturbed) populations. We also find that habitat contrast, body mass, clutch size and diet are the best predictors of direction and magnitude of tolerance of human disturbance. When we focused only on bird populations from the rural–urban habitat contrast, however, we find that body mass, diet and habitat openness are the best predictors of direction and magnitude of tolerance of these populations. Our results help to identify species that are potentially more vulnerable to increasing human disturbance and provide evidence-based guidance to future conservation actions.

## Results

### Overall results

**Figure 1:**
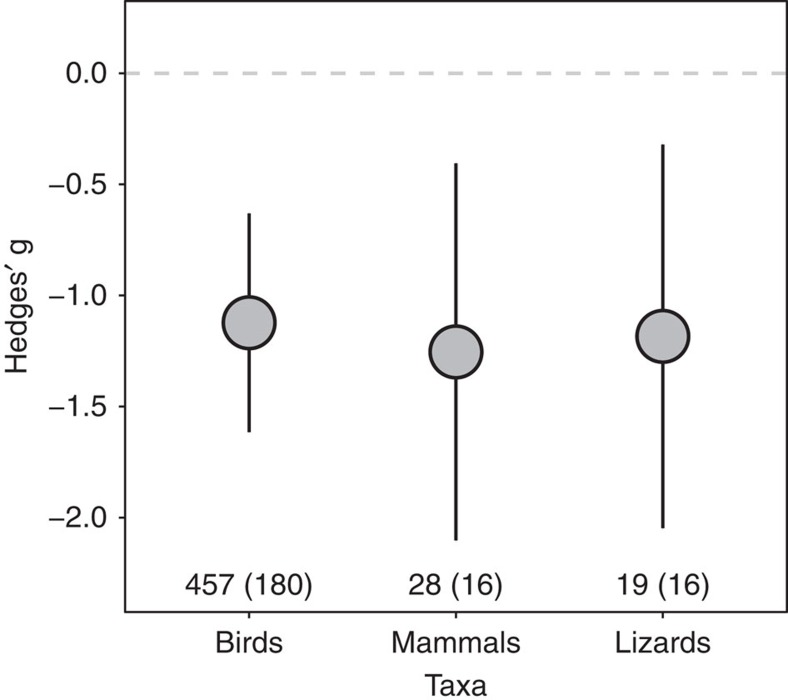
Mean (±95% confidence interval) effect sizes (Hedges' g) by taxa which compare the difference between flight initiation distances of populations under high and low human disturbance. Number of effect sizes and species (the latter in parenthesis) sampled by taxa is shown in the bottom of figure. Horizontal dashed line indicates zero effect size. Negative values illustrate tolerance of human disturbance.

In general, lizards, mammals and birds became equally more tolerant of human approaches with increasing human presence (*Q*_b_=0.09, df=2, *P*=0.96; Fig. 1 and Supplementary Data 1). Importantly, a regression between the mean and standard deviation of raw FID values showed that our effect size estimates genuinely reflect the magnitude of the mean FID differences of populations in areas of low versus high human disturbance (that is, they were not biased by any potential difference in variance of FIDs as a function of human disturbance level; Supplementary Fig. 1). Using *I*^2^, an index of heterogeneity among effect sizes^15^, we found substantial variation both in the meta-analysis of the three major taxa (*I*^2^_total_=91.44%, *I*^2^_between-study_=46.09%, *I*^2^_phylogeny_=10.15%, *I*^2^_within-study(residuals)_=35.20%) and in the birds-only meta-analysis (*I*^2^_total_=89.51%, *I*^2^_study_=36.87%, *I*^2^_phylogeny_=13.90%, *I*^2^_residual_=38.74%). This amount of heterogeneity justified our further exploration of the covariates. There was little evidence that our conclusions were affected by publication bias, where studies with low sample sizes are more prone to rejection because their higher probability of finding non-significant effects^16^ (Egger's regression; all taxa: intercept=−0.22, *P*=0.243; birds-only: intercept=−0.17, *P*=0.397; Supplementary Fig. 2). Using *H*^2^, an index for phylogenetic signal^17^, we found relatively small phylogenetic signal in the species tolerance of human disturbance (all taxa: *H*^2^=11.10%; birds-only: *H*^2^=15.53%). In fact, a separate set of analyses with meta-analytic models using studies and species as random factors, but not controlling for the phylogeny, yielded the same conclusions (Supplementary Figs 3–6, Supplementary Tables 1 and 2 and Supplementary Data 2 and 3).

### Model selection using all bird species

**Figure 2:**
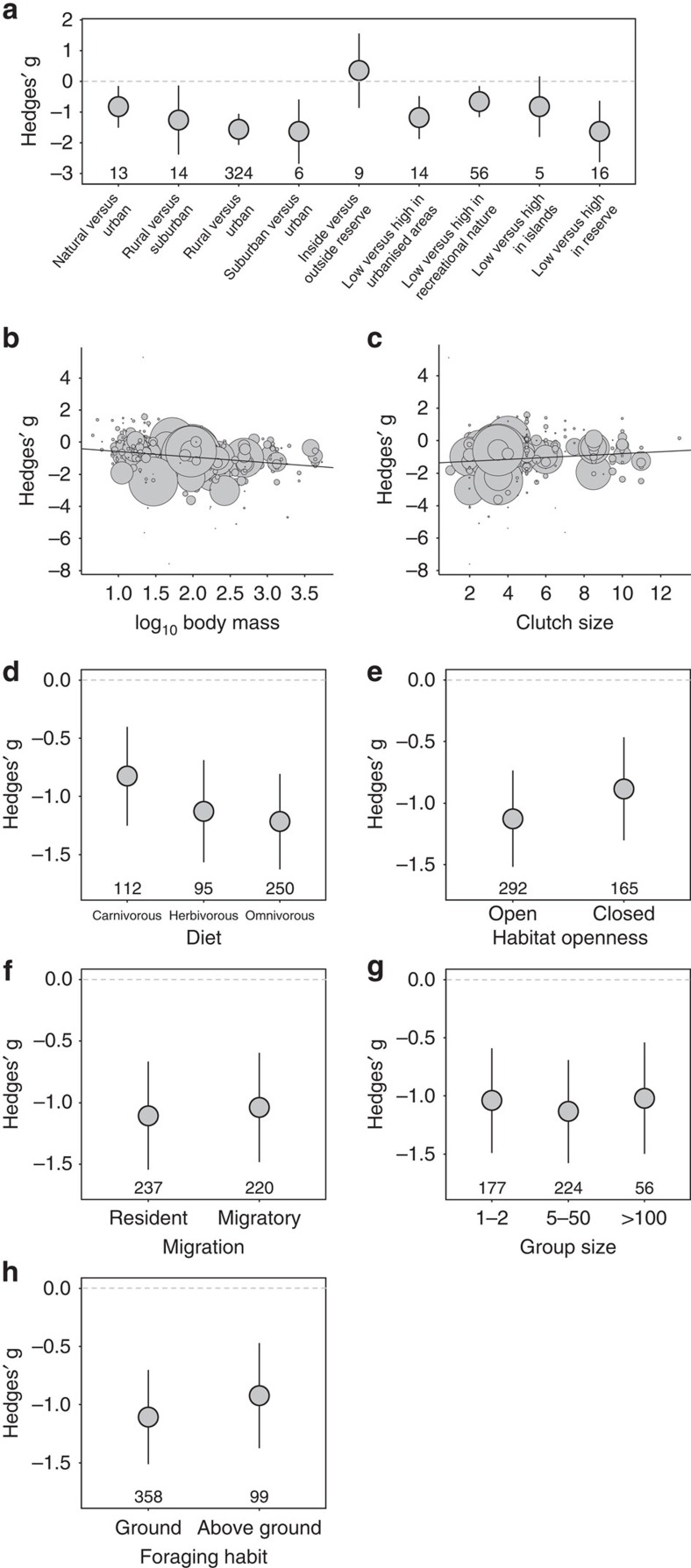
Effects of eight predictors on the effect sizes (Hedges' g) of bird's tolerance of human disturbance using the full data set (180 species, 457 effect sizes). Negative effect sizes show that species tolerate human disturbance, whereas positive effect sizes show that species are intolerant of human disturbance. Predictors are presented in their order of importance in explaining bird's tolerance (or intolerance) to human disturbance. (**a**) Type of habitats contrasted (contrasts are presented as habitat with ‘low versus high' human disturbance). Habitat contrasts presented as ‘low versus high in' refer to contrast between populations experiencing low and high human disturbance within a given habitat type. (**b**) Body mass (g). (**c**) Mean clutch size per reproductive period. (**d**) Diet. (**e**) Habitat openness. (**f**) Migration. (**g**) Group size. (**h**) Foraging habit. Horizontal dashed line indicates zero effect size. Different sizes of symbols in **b** and **c** reflect differences in sample size. The error bars illustrate 95% confidence intervals. The number of effect sizes in each categorical level is shown in the bottom of figures.

The best predictors of bird response to human disturbance were habitat contrast, body mass, clutch size and diet (Table 1 and Supplementary Data 4). Magnitude and direction of birds responses varied according to the habitat contrast (*Q*_b_=20.34, df=8, *P*=0.009; Fig. 2). Birds in urbanized environments (that is, rural versus urban populations, and suburban versus urban populations) showed the greatest tolerance of humans (Table 1 and Fig. 2). Overall, the magnitude of the FID difference (that is, the effect sizes) of populations under low versus high human disturbance was lower, albeit marginally significant, in contrasts made ‘within' a same habitat type (for example, low versus high human disturbance in islands) than in contrasts made ‘between' habitat types (for example, rural versus urban areas; *Q*_b_=3.70, df=1, *P*=0.054). Larger birds were more tolerant of human disturbance than smaller birds (Table 1 and Fig. 2). Species that produced more eggs were less tolerant of human disturbance than those that produced fewer eggs (Table 1 and Fig. 2). Herbivorous and omnivorous species were more tolerant than carnivorous species (Table 1 and Fig. 2).

### Model selection using birds from the rural–urban contrast

**Figure 3:**
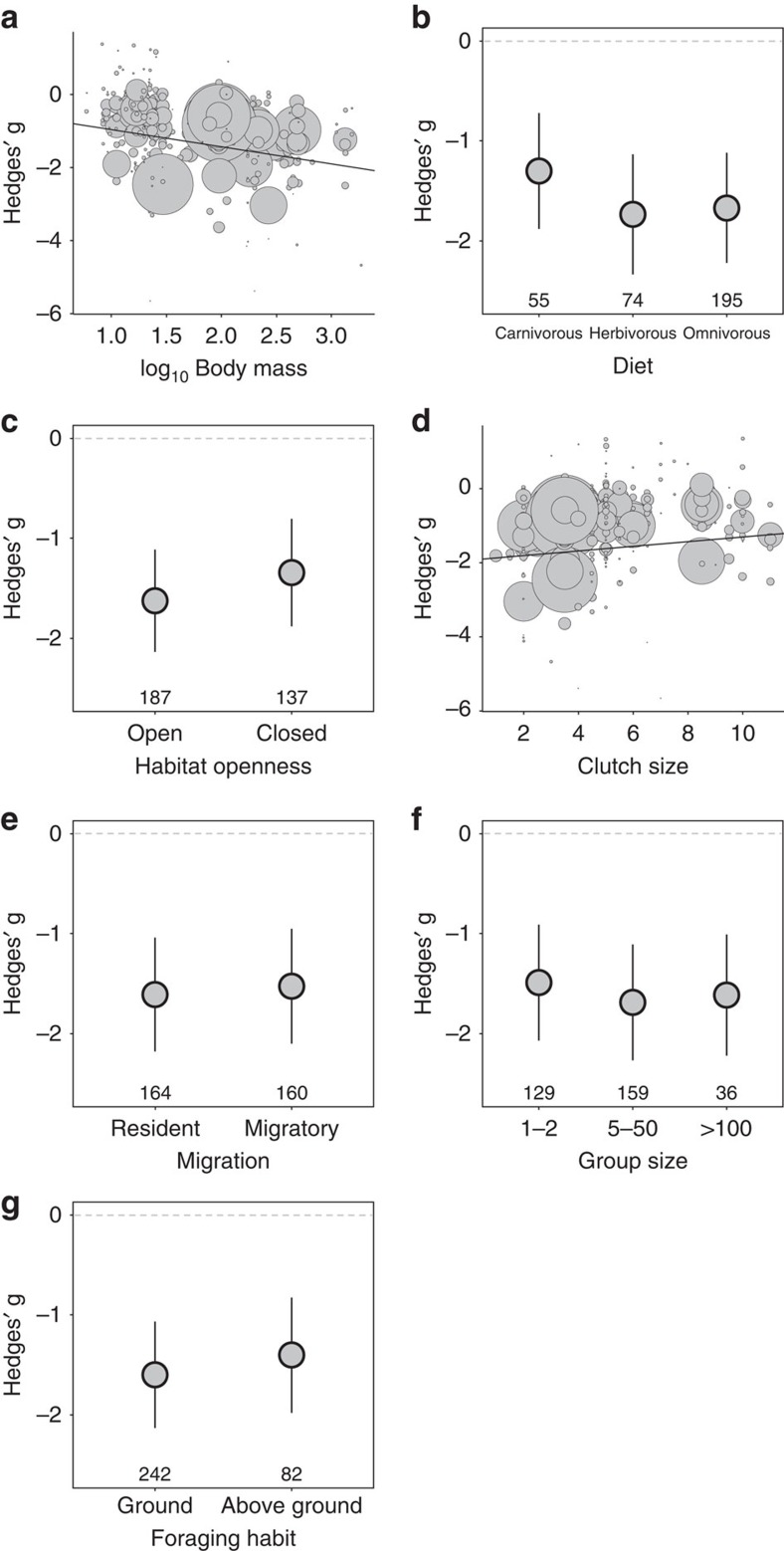
Effects of seven predictors on the effect sizes (Hedges g) of bird s tolerance of human disturbance from populations of rural-urban habitat contrast (103 species, 324 effect sizes). Negative effect sizes show that species tolerate human disturbance, whereas positive effect sizes show that species are intolerant of human disturbance. Predictors are presented in their order of importance in explaining bird's response to human disturbance. (**a**) Body mass (g). (**b**) Diet. (**c**) Habitat openness. (**d**) Mean clutch size per reproductive period. (**e**) Migration. (**f**) Group size. (**g**) Foraging habit. Horizontal dashed line indicates zero effect size. Different sizes of symbols in **a** and **d** reflect differences in sample size. The error bars illustrate 95% confidence intervals. The number of effect sizes in each categorical level is shown in the bottom of figures.

When we focused only on bird populations from the rural–urban habitat contrast, we found a slightly different rank order of importance of the best predictors (Table 1 and Supplementary Data 5). Once again, body mass was the most important species trait that explained variation in tolerance of human disturbance. However, both the larger regression coefficient and greater importance in relation to the other traits suggest that the effect of body mass on tolerance was even greater in the rural–urban habitat contrast (Fig. 3 and Table 1). Diet was the second most important trait explaining species tolerance in the rural–urban habitat contrast (Fig. 3 and Table 1). Species originally from open habitats were more tolerant than those from closed habitats (Fig. 3 and Table 1). Clutch size was substantially less important in explaining species tolerance in rural–urban habitat contrast (Fig. 3 and Table 1); an effect not explained by potential reductions in clutch size variability in urban species because of reduction in their body sizes (Supplementary Fig. 7). Similar to the analysis using the full avian data set, migration, group size and foraging habit were relatively less important in explaining variation in tolerance of human disturbance in the rural–urban habitat contrast (Fig. 3 and Table 1).

## Discussion

We found the habitat type contrasted in populations under low and high human disturbance (that is, the habitat contrasts) was one of the main drivers of the degree to which birds tolerate human disturbance. Rural versus urban and suburban versus urban populations had the largest effect sizes among the habitat contrasts, indicating that urbanized birds are those with the highest degree of tolerance of human disturbance. A previous study showed that time since urbanization was positively associated with the degree of tolerance in birds^18^, which could help to explain further variation in human tolerance within urban places. However, the lack of additional data^18^ prevents further tests of the effect of time since urbanization as a predictor of tolerance of human disturbance. Although sample sizes were admittedly smaller for the other habitat contrasts, the effect sizes of all contrasts (except inside versus outside reserves) were in the same direction, suggesting that different types of human exposure act generally increasing tolerance of populations. It is essential to realize that some species might be absent from certain habitat comparison because they are unable to survive in habitats with high human presence (for example, in urban areas) because of their low tolerance for human activity. Therefore, a more detailed examination of variation in human tolerance among habitats types would require more sampling in most habitats, a historical record of human or bird invasions^18^, and a systematic measurement of human presence (as conducted by few studies of our data set^19^,^20^). Nevertheless, our finding that FID differences were lower in contrasts within habitat types than between habitat types seems to support previous studies that showed a positive relationship between human exposure and degree of human tolerance^20^,^21^,^22^.

Based on a robust body of evidence^23^,^24^,^25^,^26^,^27^, we expected body mass to be one of the most important traits affecting how species respond to increased human disturbance. However, we did not initially expect the observed direction of the response (see Covariates in Methods). It is well established that large animals are more intolerants to human presence than small animals (that is, they have larger FID^23^,^24^,^25^,^26^,^27^), but, surprisingly, we found that large birds were those that had the greatest reduction in FID as human disturbance increased. Optimal escape theory states that animals must counterbalance both costs and benefits when making escape decisions^27^,^28^. Larger animals may suffer higher costs of flight either because they are less agile because of their size^29^,^30^ or because their energetic costs of not foraging are greater (opportunity cost^26^,^31^,^32^). Either way, we suggest that larger birds (and potentially, larger species of other taxa too) might be under intense pressure to be more tolerant of non-lethal human approaches. This finding may indicate that energetic costs of unnecessary escape are important variables driving tolerance.

There are at least two other reasons why body size may be associated with increased tolerance of humans in birds. First, larger birds may also be less likely to be killed by predators because of their body size^33^ and this reduced risk may select for increased tolerance of non-threatening humans in areas where humans are commonly encountered. Second, larger birds with relatively larger brains may have greater cognitive abilities and might be able to better assess risk^34^.

At a proximate level, our finding that carnivorous birds are less tolerant of humans may reflect a carry-over effect from their increased sensitivity to movement and thus their general degree of responsiveness^23^, or it may reflect the reduced foraging efficiency of carnivorous birds foraging around humans if their prey are similarly disturbed by humans. Carnivorous birds may also be persecuted because they compete with humans for prey, and, if so, may have been selected to be less tolerant of humans. Regardless of the mechanism, the evidence accumulated to date, and summarized in our meta-analysis, suggests that carnivorous birds are likely to be particularly vulnerable to human disturbance.

Animals in open habitats should be better able to monitor predator behaviour once they are detected, and to track risk dynamically^34^. Based on the ability to better track predation risk, we expect that animals in open habitats to be better at learning not fear encounters with non-threating humans than animals living in visually closed environments where sightings are either brief or interrupted^34^. Human-altered habitats, such as urban places, are often characterized by having reduced vegetative cover. Species naturally living in open habitats might suffer less in urbanized habitats once vegetation cover is reduced in cities, and consequently they might better tolerate humans. This may explain why habitat use (specifically, regarding to habitat openness) was a trait particularly important in explaining variation in tolerance in urbanized birds. As an extension of this reasoning, it is also probable that nature-based wildlife tourism may be less harmful to animals in relatively open habitats because humans can be monitored and animals would only escape when they assess a high risk of predation (but see ref. 35). Future studies are necessary to confirm this hypothesis.

Our meta-analysis shows that bird species that produce smaller clutches are more tolerant of human disturbance than those that produce larger clutches. Life history theory predicts that species that invest more in their offspring should be more likely to protect that investment^36^,^37^ (but see ref. 38). If we assume that small clutch-sized species generally have larger parental investment per offspring, then adults should accept more risk to guarantee their reproductive success. Abandoning a profitable patch, in addition to the energetic cost of flight, should thus be particularly costly to these species. Therefore, there should be a pressure for small clutch-sized birds to become more tolerant of non-threatening encounters. This hypothesis is consistent with the energetic hypothesis proposed by optimal escape theory^27^,^28^.

At least three mechanisms can explain variation in tolerance as a function of human disturbance. First, recent studies have suggested that differential selection among personality types could account for increased tolerance of humans in some species, whereby bold individuals settle in areas with higher human disturbance and shy individuals settle in areas with reduced disturbance^14^,^39^. Second, increased tolerance could result from local adaptation^40^. Third, increased tolerance could result from habituation^41^,^42^. We suggest that habituation-like processes are likely to be common and important because we know that individuals are exquisitely sensitive to unexpected behaviour from humans and potential predators^27^. Indeed, we expect location-specific habituation where even a slight deviation in a predator's (or human's) routine behaviour can re-elicit fearful responses^43^ (for example, when predator appears on a different side of lake^44^ or a human steps off a well-travelled hiking trail or crosses a fence^45^). Such a pattern is entirely consistent with habituation.

Discriminating between the three competing mechanistic hypotheses to explain tolerance of human disturbance would require a set of long-term studies on marked individuals (for example, because habituation is a learning process that occurs in individuals over time^42^), in addition to population-level genetic information to investigate selection and adaptation. These data are currently unavailable for the vast majority of the species studied. However, our results show that regardless of the mechanism leading to increasing tolerance of human disturbance, its magnitude and direction, at least in birds, is influenced by a species' traits and environmental differences. The present study thus helps address an urgent need to identify species potentially more vulnerable to the threats of human expansion^10^,^46^.

The results of our systematic review and meta-analysis have implications for wildlife conservation. For example, our findings suggest that small birds may benefit more from protection that reduces human disturbance than large birds, specifically because they are less likely to become tolerant of human disturbance. In practical terms, it is possible that set-back distance zones established within protected areas^10^ are more effective for small bird species. In addition, and *contra* previous suggestions based only on the relationship between body size and FID^23^, our findings suggest that ecotourism may not be as deleterious for larger birds (and perhaps larger animals) because they are better able to tolerate human disturbance and consequently may suffer smaller population-level consequences^2^,^4^,^5^.

Despite the conservation implications discussed above, at least two points related to our results require caution. First, large birds might become more vulnerable to illegal hunting if they become tolerant of humans as a function of exposure^47^. Thus, exposure to benign human presence (for example, ecotourists) could create an ecological trap if tolerant birds later encounter hunters. Therefore, wildlife managers interested in using our results to design a management programme should also consider actions aimed to curb poaching.

Second, the bird traits correlated with greater tolerance of human disturbance are the same found among many endangered species (that is, endangered species are typically large body sized and have low reproductive rates^46^,^48^,^49^). This apparently contradictory finding can be explained by considering the source of human disturbance. Large animals with low reproductive rates are in decline mainly because of habitat loss, overexploitation by humans and by climate change^49^,^50^. Large animals require larger home ranges, have reduced recruitment and are often hunted by humans for food and recreation^46^,^48^,^49^. Our results focus on behavioural responses to non-lethal human disturbance (that is, tolerance), which may influence a species' ability to live successfully with humans^2^,^4^,^5^,^6^,^7^,^42^. Therefore, although our findings may be used to predict bird species potentially most vulnerable to human disturbance, and to manage the impacts of increasing human presence on wildlife, other conservation actions must be adopted to decelerate the alarmingly rapid loss of biodiversity^51^.

The present study represents the first effort to develop an evidence-based model of wildlife tolerance of human disturbance. We hope the results of our meta-analysis serve as a starting point to predict and manage the impacts of increasing human disturbance on wildlife and guide conservation strategies. Additional studies must be conducted to understand the mechanisms that promote tolerance of human disturbance, because this mechanistic understanding should help us design more effective management strategies^10^,^52^. Also, studies are necessary to determine visitation rates that maximise the development of tolerance of humans in wildlife. Ultimately, it is evidence-based actions that will help reduce biodiversity loss in a rapidly urbanizing world.

## Methods

### Literature search and selection criteria

We first compiled all studies that cited or were cited by two key reviews^28^,^31^. We searched for studies published before 31 July 2014 on Web of Knowledge, Scopus and Google Scholar databases with the key words ‘flight initiation distance', ‘flight distance', ‘escape distance', ‘approach distance', ‘flushing distance' and ‘response distance'. The references cited in these studies were also examined. Our criteria to include a study were that FID of a given species (measured *sensu*^27^) had to be collected in areas of low and high human presence (=human disturbance). We followed the criteria used by authors to categorize each area as a function of its degree of disturbance. Hunted populations were not included in our data set. A PRISMA diagram describing our literature search and the detailed reasons for exclusion of studies are available in Supplementary Fig. 8 and Supplementary Table 3, respectively. Our final data set consisted of 504 effect sizes from 75 studies across 212 species distributed in three major taxa: birds, mammals and lizards (data available in Supplementary Data 1).

### Estimating effect sizes

To quantify species response to disturbance, we used the effect size metric Hedges' g^11^; a bias-corrected measure of standardized mean differences, which does not overestimate the magnitude of effect when sample size is small^11^. For each species, we compared the mean FID of populations in areas of higher human disturbance with those in areas of lower human disturbance. These FID comparisons were restricted to populations of species studied in a same study. In our data set (Supplementary Data 1), positive effect sizes indicate sensitization, whereas negative values indicate tolerance of human disturbance. When mean, variance and sample size of FIDs were not provided in a paper, we estimated Hedges'g from the statistical results (*t*, F, *χ*^2^, *Z* and *P*)^53^. We directly contacted several authors for missing data (see Acknowledgements for details). Importantly, recent studies have shown that the starting distance of an approaching person (that is, animal–human distance when the approach begins) can affect FID^27^. However, the potential effects of starting distance on FID were controlled in most effect sizes either because studies used a fixed starting distance among experiments (for example, starting distance fixed in 30 m) or because starting distance was controlled analytically by using it as a covariate in statistical models, or because we used the marginal means for those studies that provided them or those studies in which we were able to obtain the raw data (see Supplementary Data 1 for details).

### Meta-analysis

We used multi-level mixed-effects meta-analysis^17^ to test for both mean effect sizes and the importance of our predictors. We controlled for phylogenetic and study non-independence by including phylogeny (Supplementary Fig. 9) and study identity as random-factors in our models^17^. Although we also have multiple estimates per species in our data set (Supplementary Data 1), a model selection approach showed that the inclusion of ‘species identity' as an additional random-effect did not improve our models (Supplementary Table 4). Phylogenies of birds, mammals and lizards and how they were combined to test for difference among these taxa is described in Supplementary Methods 1. The mean effect sizes (that is, mean of the effect sizes weighted by the inverse of their variance) were considered significant if their 95% confidence intervals did not include zero^53^. We used the between-groups heterogeneity statistic (*Q*_b_) to test for significant difference between mean effect sizes^53^,^54^.

We used *I*^2^ index as a measure of heterogeneity in the effect sizes in which the value represents the proportion of total variation in data that is not sampling error (0%—all sampling error; 100%—no sampling error)^15^. We used an extended version of *I*^2^ that partitions the total heterogeneity among different sources: variation explained by study identity, by phylogenetic effect and by the residual variation (that is, that remaining to be explained by the predictor variables^17^). We calculated the degree of phylogenetic signal in our effect size estimates using the phylogenetic heritability index^17^, *H*^2^, which is the variance attributable to phylogeny in relation to the total variance expected in the data. When the unit of analysis is species, *H*^2^ is equivalent to Pagel's *λ* (ref. 55), in which higher values are associated with stronger phylogenetic signals. Primary studies can suffer from publication bias, where studies with low sample size are more prone to be rejected because of their higher probability of not finding significant effects^16^,^53^. We checked for publication bias using Egger's regression^16^, in which intercepts significantly different from zero suggest potential publication bias. To overcome the non-independent nature of our data, we applied the Egger's regression test on the meta-analytic residuals^17^. Analyses were conducted using the *metafor*^54^ R package v.1.9-4.

### Covariates

The large number of observations in birds permitted us to investigate whether certain variables were potentially important predictors of tolerance of human disturbance. Based on previous findings in the literature and our own hypotheses, we collected information on eight variables. Seven were associated with a species' morphology, life-history and natural history traits. These data were obtained from Del Royo *et al*.^56^ (see Supplementary Data 1 for details). The eighth variable, the habitat contrast, describe the type of habitats contrasted in populations under low and high human disturbance. These data were extracted from each surveyed paper. Importantly, for these covariates, multi-collinearity was not an issue (variance inflation factor<1.50, below the suggested threshold^57^ of 3; see also correlation matrix in Supplementary Table 5). Below we justify the use of each variable and our predictions concerning their effect on bird response.

*Body mass*. A substantial amount of empirical evidence shows that large animals are less tolerant of human approaches—they generally flush at a greater distance from humans^23^,^24^,^25^,^26^,^27^. There are two non-exclusive main hypotheses to explain this response: (i) large animals are generally less maneuverable^29^,^30^ or (ii) large animals suffer a greater opportunity cost of not foraging because of their greater absolute metabolic needs^27^,^32^. Thus, we hypothesized that large animals would tolerate less human disturbance by showing larger positive effect sizes than small animals. Body mass was measured in grams and log_10_ transformed before analysis.

*Group size*. Three models of predation risk assessment predict a declining risk of predation as group size increases^58^,^59^,^60^. If predation risk decreases with group size, individuals in larger groups might thus tolerate closer approach. Moreover, if tolerance of non-lethal human disturbance is a socially transmitted behaviour, one could expect the effect of social transmission to be enhanced in larger groups^61^. Therefore, group size should be expected to influence tolerance of human disturbance by increasing the tolerance as group size increases. Following Burish *et al*.^62^, we coded species into three categories: alone or in pairs, in groups of 5–50 individuals, in groups of >100 individuals.

*Habitat openness*. Animals in open habitats can simultaneously detect predators at a greater distance and might have to travel a longer distance to reach protective cover. A recent study showed that birds originally from open habitats tend to delay the flight when compared with birds from closed habitats^34^. Moreover, highly altered habitats, such as urban and suburban places, usually have reduced vegetative cover. We thus hypothesized that species naturally living in open habitats would better tolerate humans because they would suffer less as vegetation cover was reduced. Either way, habitat openness might influence FID and must be accounted for if we are to isolate human disturbance effects. We categorized species as being originally from open habitats (for example, uplands and grassland) or closed habitats (for example, dense forests and woodlands).

*Foraging habit*. Prior work has shown that in areas of human disturbance, birds may place their nests higher in trees^63^,^64^ suggesting that being in trees affords enhanced safety. However, a previous study looking at the effects of height in a tree on FID found either no effects or found that birds that were higher in trees initiated flight at greater distances^65^. Our prediction is that species that typically forage on the ground would be under greater pressure to tolerate humans to minimize the opportunity cost of resuming foraging after a potentially unwarranted escape. We thus classified species as typically foraging on the ground versus species typically foraging above of ground level (for example, in trees or catching aerial insects while flying).

*Diet.* Birds that eat live prey, particularly carnivorous raptors, have especially good visual acuity and motion sensitivity^66^. Given this sensitivity, birds that eat live prey are disturbed at greater distances^23^. This increased sensitivity to disturbance could select for tolerance if they are better to learn that humans are not a threat. Alternatively, it could also reflect the possibility that foraging efficiency is reduced because their prey are similarly disturbed by humans. In the latter case, we might expect that these species would be less likely to tolerate the on-going disturbance. We categorized species as carnivorous, herbivorous or omnivorous.

*Migration*. Birds that migrate are exposed to a greater variety of habitats and might be selected to rapidly learn to assess predation risk in new habitats. Under this scenario, we expected that these species would be more likely to tolerate increased disturbance. We coded species as resident or migratory.

*Clutch size*. Like body size, clutch size is a life history trait that reflects energetic investment, and therefore need^37^. In general, we might expect that birds that produce fewer eggs per reproductive period might be more energetically stressed than those that produce more eggs because they have larger parental investment per offspring^36^. In this case, small clutch-sized species would tolerate closer approach because of the greater opportunity costs associated with flight^27^,^28^. Alternatively, enhanced energetic needs might select for small clutch-sized species to not tolerate the on-going monitoring costs associated with disturbance^67^ and may thus select them to move off and forage in areas without disturbance, resulting in a lower tolerance of these species. Either way, clutch size must be accounted for to isolate the effects of human disturbance on flight. We used the estimates of the number of eggs per reproductive period. Because there was a low correlation between clutch size and body mass (Supplementary Table 5), clutch size effects were not corrected by species body mass in our analyses.

*Habitat contrast*. The nine habitat contrasts were: (i) natural versus urban area, (ii) rural versus suburban areas, (iii) rural versus urban area, (iv) suburban versus urban area, (v) inside versus outside reserve, (vi) low versus high human disturbance in urbanized areas, (vii) low versus high human disturbance in recreational nature (for example, beaches, ski areas and other tourist locations), (viii) low versus high human disturbance in islands and (ix) low versus high human disturbance in reserve. We used these habitat contrasts either because of their difference in human disturbance degree or because a particular characteristic of habitat is expected to influence animal's tolerance of humans. Specifically, contrasts between natural, rural, suburban and urban populations were tested because they represented increasing human presence and thus a differential human tolerance is expected as a function of frequency of exposure to humans^20^,^21^,^22^. Previous studies have shown that even subtle temporal or spatial change in human disturbance within a given habitat type may triggers changes in animal's tolerance of humans^27^,^41^,^42^,^43^,^44^,^45^, justifying our exploration of the contrasts between low versus high human disturbance within a same habitat type (levels vi–ix). Overall, we expect a lower FID difference (that is, tolerance) in comparisons within than between habitat types. Recreational areas, islands and protected areas (reserves) were tested separately because their marked difference in the pattern of human disturbance. Because island populations often have reduced predation risk compared with mainland populations^68^, we expected either none or a small difference in FIDs of different populations found on islands. Animals living in natural areas with tourism may be more responsive to humans because they commonly experience seasonality in human disturbance (for example, visitation only in summer or winter). Beyond temporal variation, populations inside and outside protected areas may suffer marked spatial variation in human disturbance, as well as potential lethality associated with human presence (for example, individuals in protected area may feel safer). This spatial variation in human tolerance may also occur in populations ‘within' protected areas if the frequency of human visitation varies in the protected area. Importantly, our habitat contrasts were restricted to comparisons between populations of species tested in the same study. As explained in the *Meta-analysis* section, the variation among studies was controlled for by using study identity as a random factor in our models.

### Multi-model inference

We used a multi-model inference approach based on Akaike's criteria corrected for small sample size to estimate the relative importance of the predictor variables^12^. To calculate the importance of each predictor, we first assessed the relative strengths of each candidate model by calculating its Akaike weight; analogous to the probability of that model is the best model. A constant term (intercept) was included in all models. In sequence, we estimated the importance of a predictor by summing the Akaike weights of all models in which that candidate variable appeared, which can be interpreted as the probability that a particular predictor is a component of the best model, which allowed us to rank predictors in order of importance^12^. We used a model averaging approach to estimate model parameters^12^. Multi-model analyses were conducted using the *MuMIn*^69^ R package v. 1.14.0.

## Additional information

**How to cite this article:** Samia, D. S. M. *et al*. Increased tolerance to humans among disturbed wildlife. *Nat. Commun.* 6:8877 doi: 10.1038/ncomms9877 (2015).

## Supplementary Material

Supplementary Information Supplementary Figures 1-9, Supplementary Tables 1-5, Supplementary Methods and Supplementary References

Supplementary Data 1 Complete data set

Supplementary Data 2 Model selection of all birds: phylogenetic effect not controlled

Supplementary Data 3 Model selection of rural-urban birds: phylogenetic effect not controlled

Supplementary Data 4 Model selection of all birds: phylogenetic effect controlled

Supplementary Data 5 Model selection of rural-urban birds: phylogenetic effect controlled

## Figures and Tables

**Table 1:** Summary of the multi-model inference conducted to explain avian responses to human disturbance.

**Predictor**	**Levels**	**Estimate**	**s.e.**	**Importance**
*All birds (180 species, 457 effect sizes)*				
Intercept		0.736	0.599	
Habitat contrast				
	Natural versus urban	−0.941	0.632	1
	Rural versus suburban	−1.438	0.762	—
	Rural versus urban	−1.689	0.589	—
	Suburban versus urban	−1.690	0.740	—
	Low versus high in urbanized areas	−1.274	0.635	—
	Low versus high in recreational nature	−0.684	0.593	—
	Low versus high in islands	−0.894	0.717	—
	Low versus high in reserve	−1.622	0.723	—
Body mass		−0.315	0.075	0.98
Clutch size		0.052	0.021	0.93
Diet				
	Herbivorous	−0.232	0.131	0.91
	Omnivorous	−0.260	0.105	—
Habitat openness		0.182	0.091	0.83
Migration		0.127	0.080	0.70
Group size				
	5–50 Individuals	−0.060	0.088	0.51
	>100 Individuals	0.097	0.138	—
Foraging habit		−0.007	0.109	0.41
				
*Rural versus urban birds (103 species, 324 effect sizes)*				
Intercept		−0.662	0.309	
Body mass		−0.403	0.088	0.99
Diet				
	Herbivorous	−0.376	0.164	0.88
	Omnivorous	−0.244	0.130	—
Habitat openness		0.215	0.105	0.85
Clutch size		0.035	0.025	0.64
Migration		0.123	0.095	0.62
Group size				
	5–50 Individuals	−0.100	0.108	0.56
	>100 Individuals	0.091	0.176	—
Foraging habit		0.079	0.130	0.46

Results are shown from both a meta-analysis using the full data set (all birds) and a meta-analysis focusing on the contrast between rural and urban populations. Values are average coefficients of models (estimate), their associated standard error (s.e.), and the importance of each factor in explaining species responses to human disturbance (the closer than 1, the most important the factor). Habitat contrasts presented as ‘low versus high in' refer to contrast between populations experiencing low and high human disturbance within a given habitat type.
