# Supplementary material for: Increased tolerance to humans among disturbed wildlife
Source: Nat Commun. 2015 Nov 16;6:8877. doi: 10.1038/ncomms9877 (PMC4660219; doi:10.1038/ncomms9877)
Supplement: Supplementary Information — Supplementary Figures 1-9, Supplementary Tables 1-5, Supplementary Methods and Supplementary References [file ncomms9877-s1.pdf]

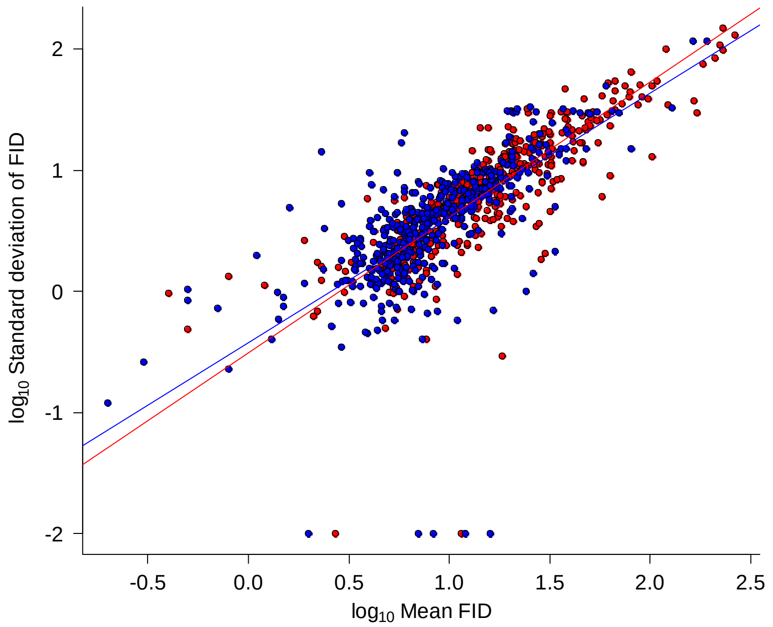

Supplementary Figure 1. Relationship between mean and standard deviation of raw flight initiation distance (FID) of populations in low (red) and high (blue) human disturbance. Whereas we found a significant positive relationship between mean and standard deviation of FIDs in populations from both disturbance levels (Low human disturbance: intercept = –0.393, b = 1.024, P < 0.001; High human disturbance: intercept = –0.353, b = 0.957, P < 0.001), the interaction between mean FID and disturbance level was not significant (b = 0.067, P = 0.269). The positive relationship between mean and standard deviation of FID is caused by an envelope constraint relationship that extends to the origin (i.e. there is no variation when mean FID is zero). Therefore, animals with larger mean FIDs also typically have larger standard deviations in FID. Because Hedges'g effect sizes were calculated as the mean differences between FIDs in low and high human disturbance divided by their pooled standard deviation[1], the absence of interaction implies that our effect sizes estimates genuinely reflect the magnitude of mean FID differences (i.e. they were not biased by any potential difference in the variance of FIDs as a function of human disturbance level).

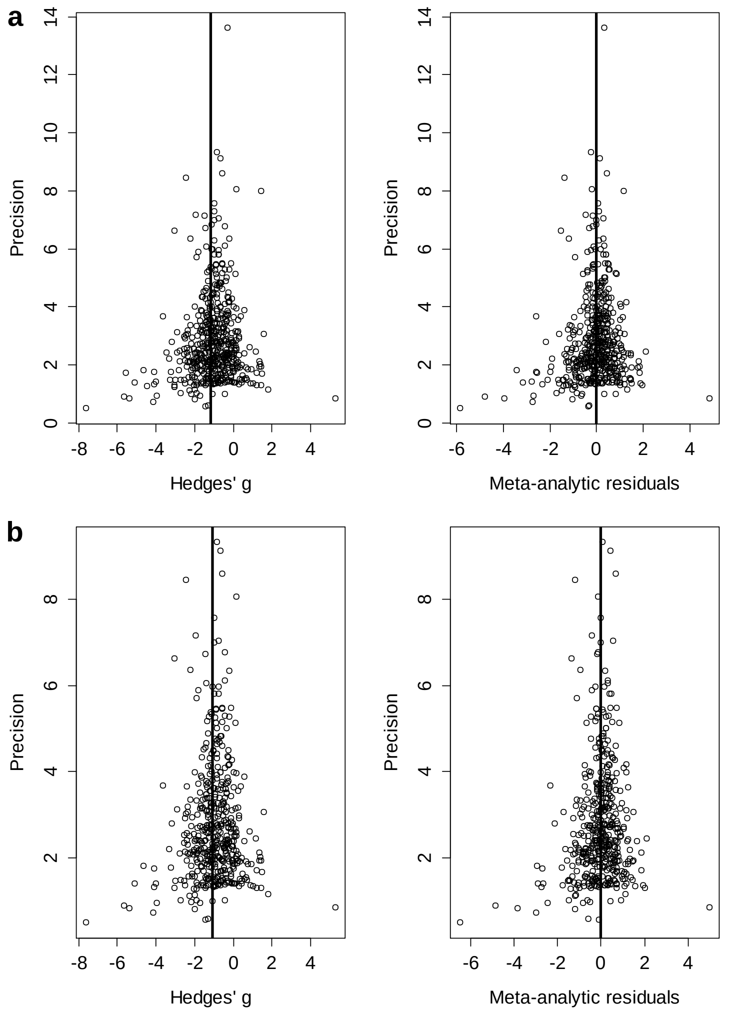

Supplementary Figure 2. Funnel plots of (a) all taxa and (b) birds-only meta-analyses made with data from meta-analytic models in which study identities and phylogeny were used as random factors. If there were an obvious bias, it would be seen by relatively more points on the left side than on the right side in the bottom half of the funnel plots (i.e. more populations with large effect size than small effect size in studies with low sample sizes). Relatively symmetrical distributions of effect sizes indicate that studies surveyed were not biased in their reporting of significant effects. This inference, based on visual assessment, was confirmed by the Egger's regression test, which found little evidence of publication bias (All taxa: intercept: -0.22, P = 0.243; Birds-only: intercept = -0.17, P = 0.397).

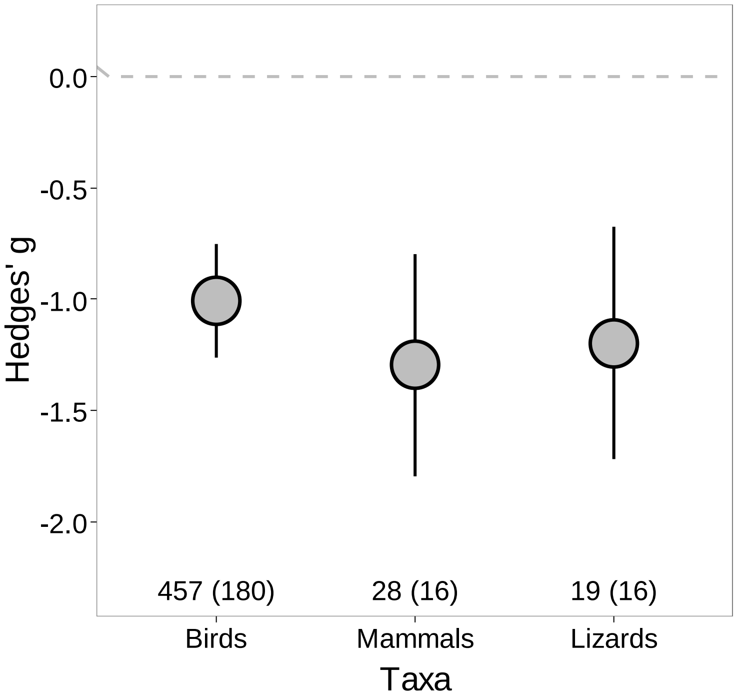

Supplementary Figure 3. Mean (±95% CI) effect sizes (Hedges' g) by taxa which compare the difference between flight initiation distances of populations under high and low human disturbance in a meta-analytic model with study identity and species as random factors (i.e. the phylogenetic dependency among species was not controlled). Number of effect sizes and species (the latter in parenthesis) sampled by taxa is shown in the bottom of figure. Horizontal dashed line indicates zero effect size. Negative values illustrate tolerance of human disturbance. Taxa did not differ in their degree of tolerance of human disturbance (Q_b = 1.21, df = 2, P = 0.54). There was substantial heterogeneity among effect sizes both in the meta-analysis including the three major taxa (I^2_{total} = 90.96%, $I^2_{between\text{-}study}$ = 46.89%, $I^2_{species}$ = 7.91%, $I^2_{with\text{-}study(residuals)}$ = 36.16 %) and in the birds-only meta-analysis (I^2_{total} = 88.99%, I^2_{study} = 40.84%, $I^2_{species}$ = 9.59%, $I^2_{residual}$ = 38.56%).

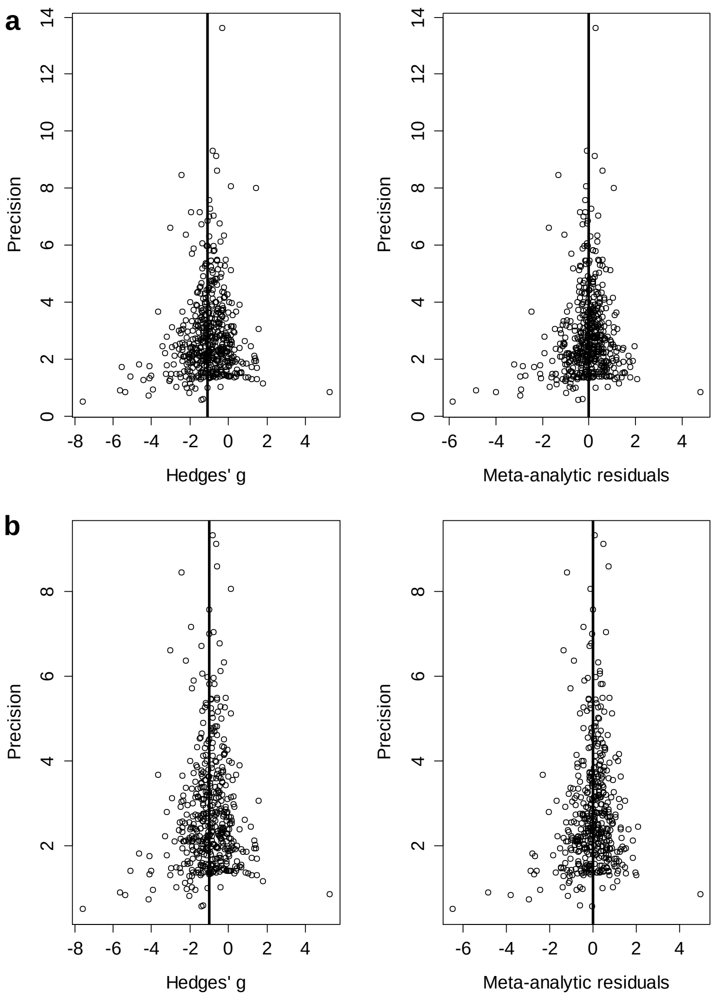

Supplementary Figure 4. Funnel plots of (a) all taxa and (b) birds-only meta-analyses made with data from meta-analytic models in which study identities and species were used as random factors (i.e. the phylogenetic dependency among species was not controlled). If there were an obvious bias, it would be seen by relatively more points on the left side than on the right side in the bottom half of the funnel plots (i.e. more populations with large effect sizes than small effect sizes in studies with low sample sizes). Relatively symmetrical distributions of effect sizes indicate that studies surveyed were not biased in their reporting of significant effects. This inference, based on visual assessment, was confirmed by the Egger's regression test that found little evidence of publication bias (All taxa: intercept: -0.21, P = 0.258; Birds-only: intercept = -0.17, P = 0.403).

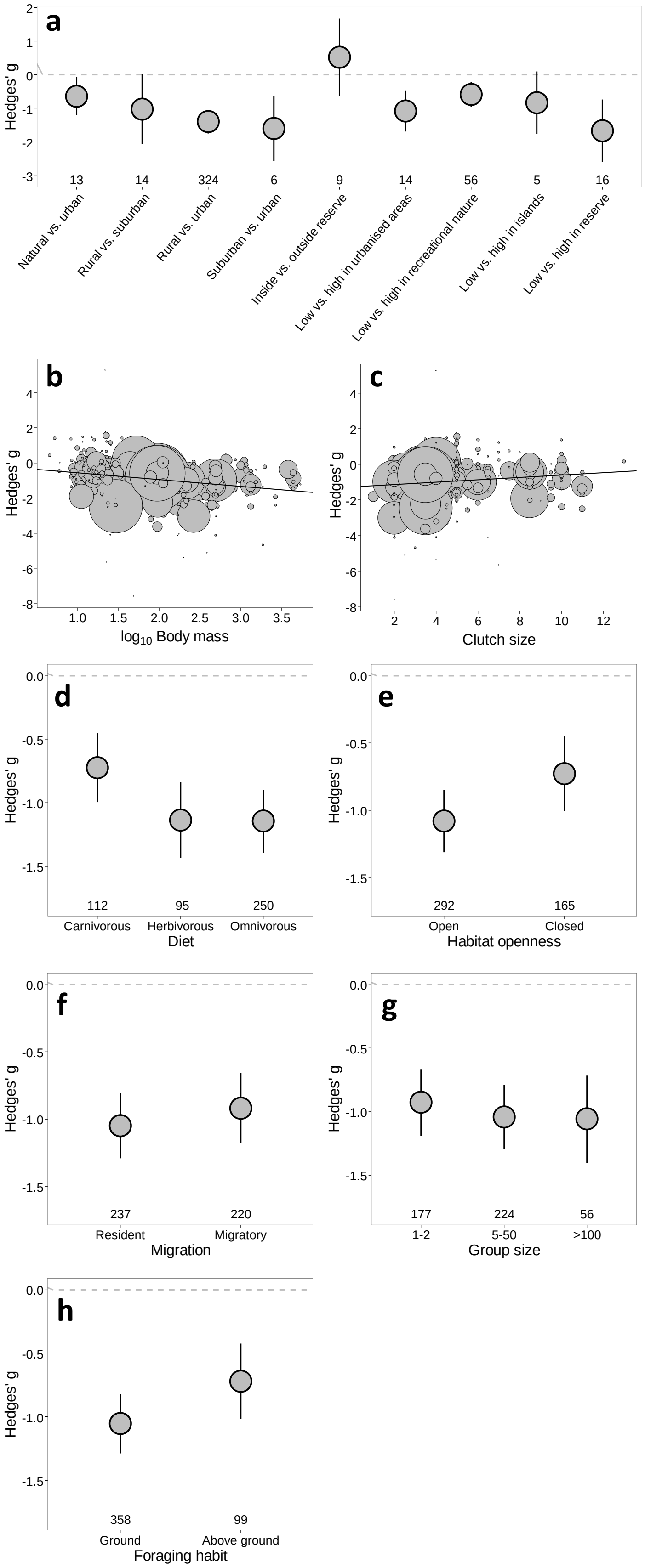

Supplementary Figure 5. Effects of eight predictors on the effect sizes (Hedges' g) of bird's tolerance of human disturbance (180 species, 457 effect sizes) in a meta-analytic model

with study identity and species as random factors (i.e. the phylogenetic dependency among species was not controlled). Negative effect sizes show that species tolerate human disturbance whereas positive effect sizes show that species are intolerant of human disturbance. Predictors are presented in their order of importance in explaining bird's response to human disturbance. **a,** Type of habitats contrasted (contrasts are presented as habitat with 'low *vs*. high' human disturbance). Habitat contrasts presented as "low *vs*. high in" refer to contrast between populations experiencing low and high human disturbance within a given habitat type. **b,** Body mass (g). **c,** Mean clutch size per reproductive period. **d,** Diet. **e,** Habitat openness. **f,** Migration. **g,** Group size. **h,** Foraging habit. The horizontal dashed line illustrates zero effect size. Different sizes of symbols in plots b and c reflect differences in sample size. The error bars illustrate 95% confidence intervals. The number of effect sizes in each categorical level is shown at the bottom of each figure.

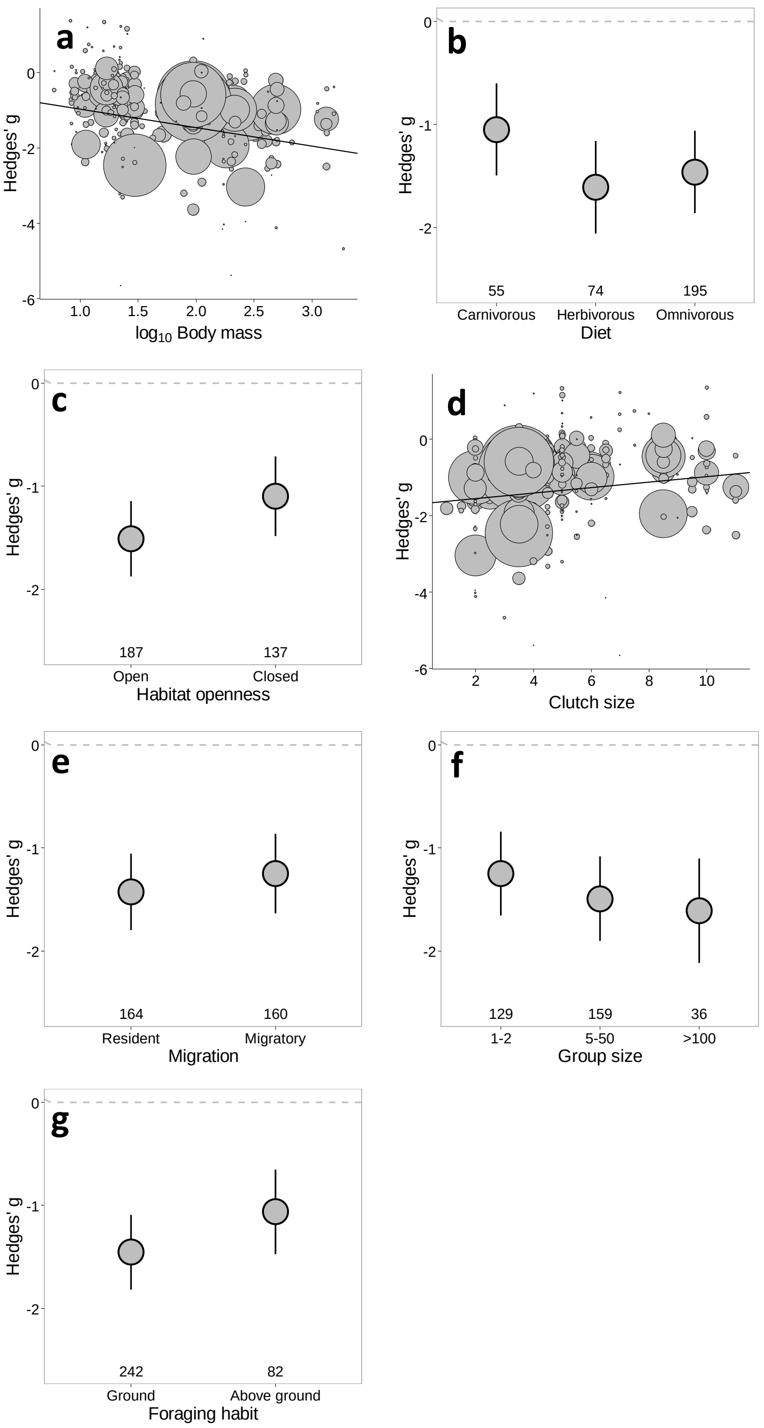

Supplementary Figure 6. Effects of seven predictors on the effect sizes (Hedges' g) of bird's tolerance of human disturbance from rural-urban habitat contrast populations (103 species, 324 effect sizes) using a meta-analytic model with study identity and species as random factors (i.e. the phylogenetic dependency among species was not controlled). Negative effect sizes show that species tolerate human disturbance whereas positive effect sizes show that species are intolerant of human disturbance. Predictors are presented in their order of

importance in explaining bird's response to human disturbance. **a,** Body mass (g). **b,** Diet. **c,** Habitat openness. **d,** Mean clutch size per reproductive period. **e,** Migration. **f,** Group size. **g,** Foraging habit. The horizontal dashed line illustrates zero effect size. Different sizes of symbols in plots a and d reflect differences in sample size. The error bars illustrate 95% confidence intervals. The number of effect sizes in each categorical level is shown at the bottom of each figure.

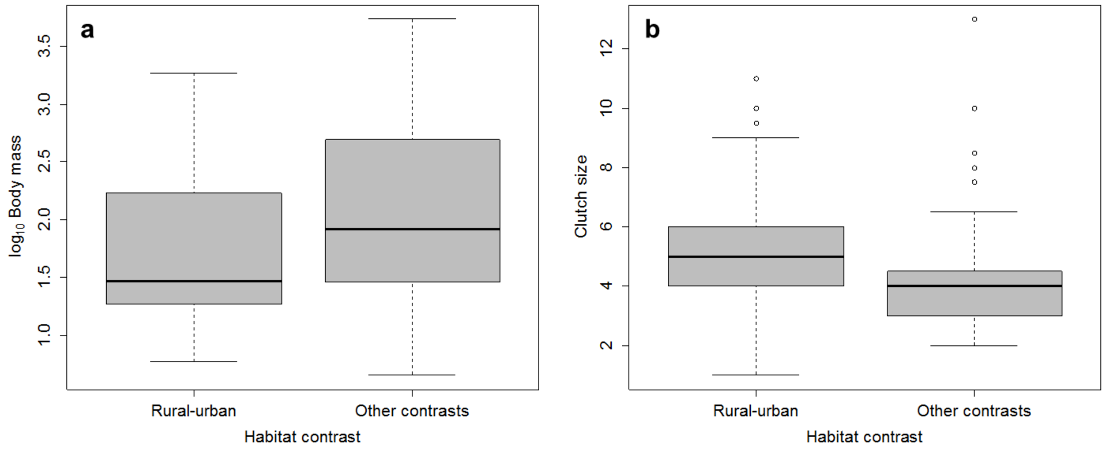

Supplementary Figure 7. Box plots showing the data distribution of (a) body mass and (b) clutch size of bird species from rural-urban habitat contrast and from other habitat contrasts. The box plots show median, quartiles, 5- and 95- percentiles and extreme values. Birds species occurring in rural-urban habitat contrast had smaller mean body masses (Unequal variance *t*-test; $t = 4.794$, df = 202.531, $P < 0.001$) and larger variance (Levene's test; $F_{1,455} = 9.493$, $P < 0.001$) than species from other habitats contrasts. These results indicate that the lower importance of clutch size in explaining bird's tolerance in the rural-urban habitat contrast was not caused by a reduction in clutch size variability in urban species, which could be caused by a reduction in their mean body size in urban places.

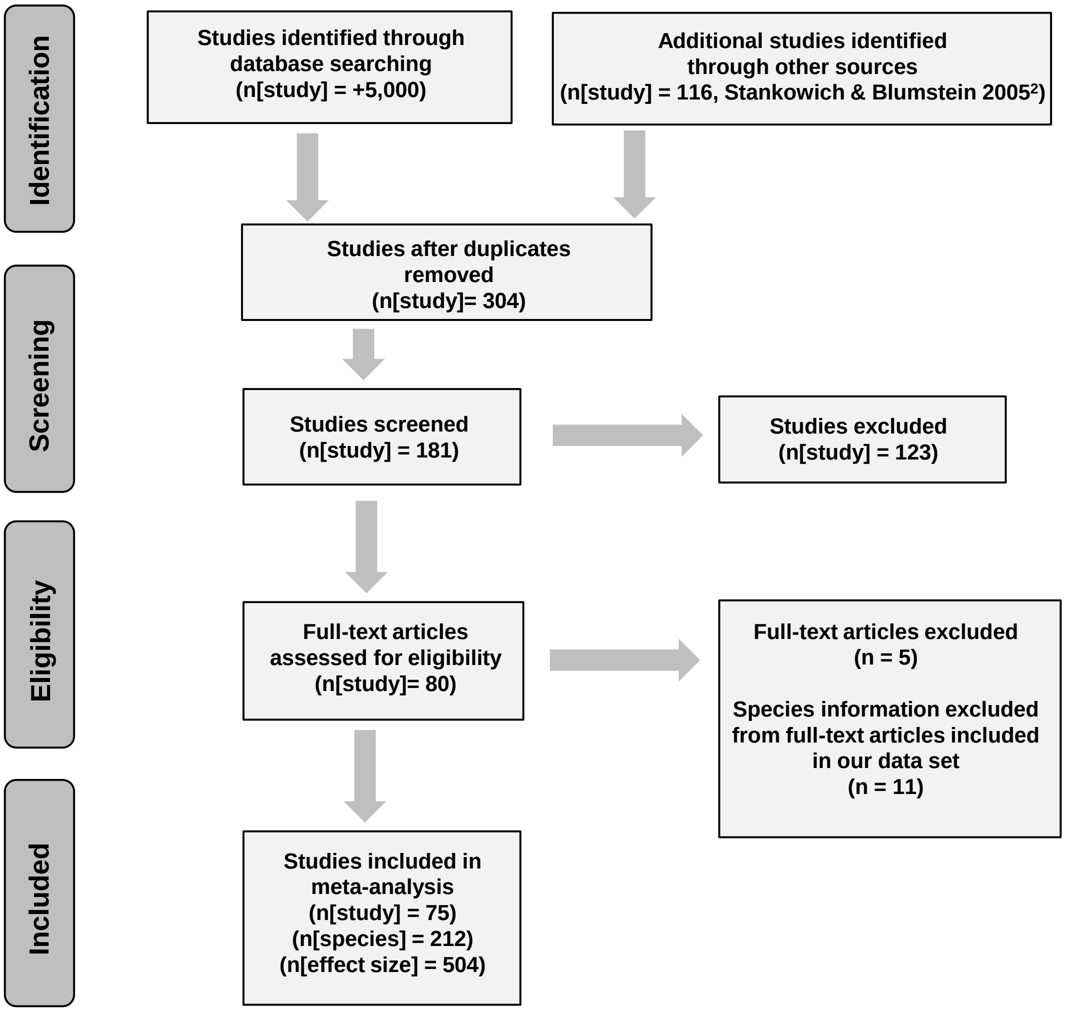

Supplementary Figure 8. PRISMA flow diagram describing the literature search.

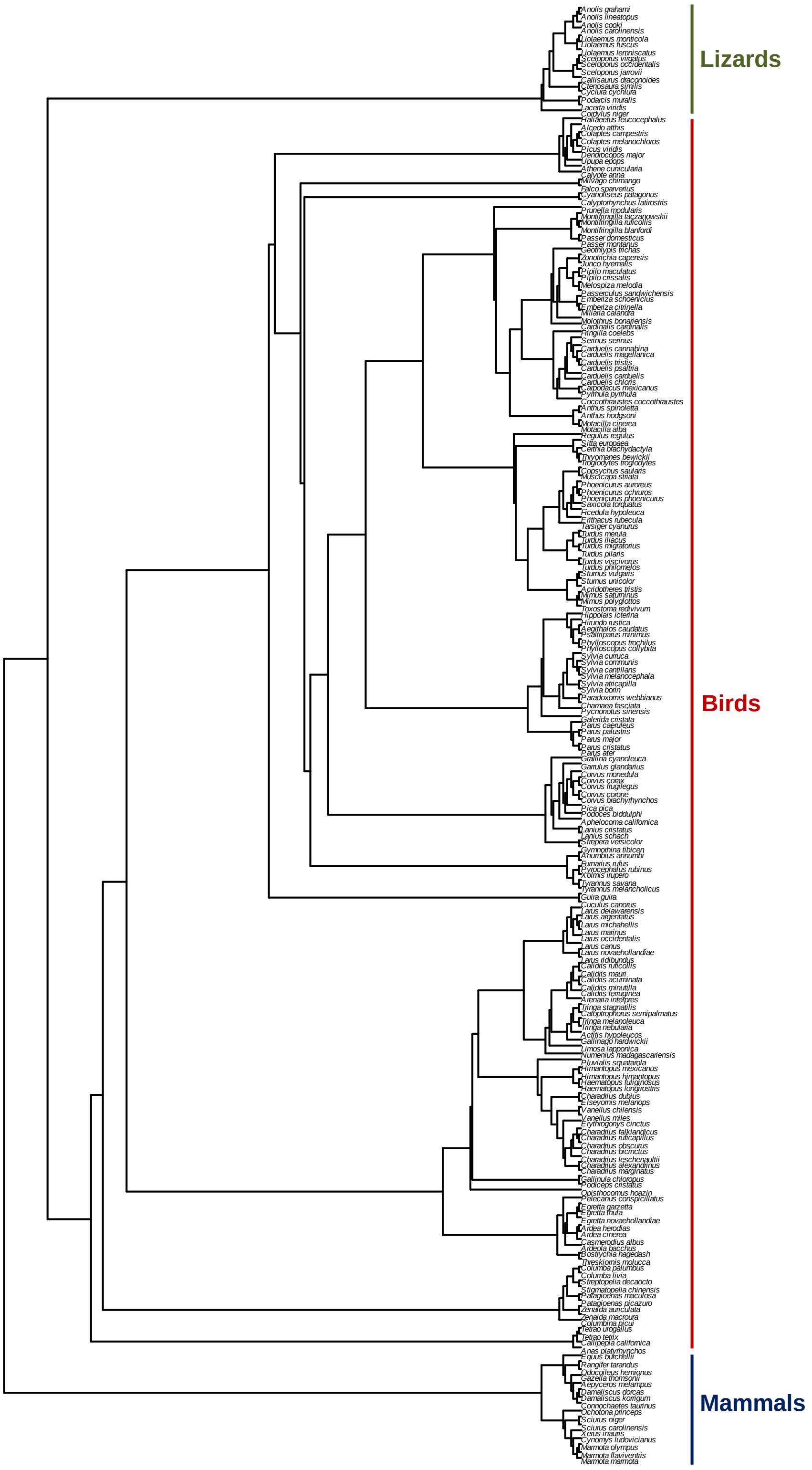

Supplementary Figure 9. Phylogeny of the 212 species included in our meta-analysis.

Supplementary Table 1. Model selection for the most parsimonious combination among two random factors

model	species	study	AICc	ΔAICc
all taxa				
model 1			1453.20	95.07
model 2	●		1445.30	87.17
model 3		●	1365.71	7.58
model 4*	●	●	1358.13	0
all birds				
model 1			1286.93	71.74
model 2	●		1276.29	61.10
model 3		●	1223.43	8.23
model 4*	●	●	1215.19	0
urban birds				
model 1			870.73	22.90
model 2	●		865.26	17.43
model 3		●	855.14	7.31
model 4*	●	●	847.83	0

The random factors are the species identity to account for multiple effect sizes per species (species) and the study identity to account for multiple effect sizes per study (study). Phylogenetic dependency among species was not included as a candidate random factor in this model selection. AICc, Akaike information criteria corrected by sample size. ΔAICc, difference in relation to the best model, i.e. the model with the lowest AICc. The best models are indicated by asterisks.

Supplementary Table 2. Summary of the multi-model inference conducted to explain avian responses to human disturbance conducted from meta-analytic models in which study identity and species were used as random factors (i.e. the phylogenetic dependency among species was not included)

Predictor	Levels	Estimate	SE	Importance
**All birds (180 species, 457 effect sizes)**				
Intercept		0.757	0.592	1
Habitat contrast	Natural *vs*. urban	-0.94	0.632	1
	Rural *vs*. suburban	-1.436	0.763	-
	Rural *vs*. urban	-1.688	0.589	-
	Suburban *vs*. urban	-1.692	0.741	-
	Low *vs*. high in urbanised areas	-1.273	0.635	-
	Low *vs*. high in recreational nature	-0.68	0.593	-
	Low *vs*. high in islands	-0.893	0.717	-
	Low *vs*. high in reserve	-1.618	0.723	-
Body mass		-0.319	0.074	1
Clutch size		0.052	0.021	0.92
Diet	Herbivorous	-0.233	0.131	0.91
	Omnivorous	-0.257	0.104	-
Habitat openness		0.181	0.091	0.82
Migration		0.127	0.08	0.71
Group size	5 – 50 individuals	-0.059	0.088	0.51
	> 100 individuals	0.098	0.138	-
Foraging habit		-0.008	0.108	0.41
**Rural *vs*. urban birds (103 species, 324 effect sizes)**				
Intercept		-0.641	0.308	1
Body mass		-0.409	0.093	1
Diet	Herbivorous	-0.383	0.17	0.88
	Omnivorous	-0.244	0.133	-
Habitat openness		0.219	0.111	0.84
Clutch size		0.035	0.026	0.63
Migration		0.124	0.098	0.62
Group size	5 – 50 individuals	-0.101	0.114	0.60
	> 100 individuals	0.117	0.185	-
Foraging habit		0.075	0.135	0.46

Results are shown both from a meta-analysis using the full data set (all birds) and from a meta-analysis focusing on the contrast between rural and urban populations. Values are average coefficients of models (estimate) and their associated standard error (SE), and the importance of each factor in explaining species responses to human disturbance (the closer to 1, the most

important the factor). Habitat contrasts presented as “low *vs*. high” mean contrast between populations with low and high human disturbance within a given habitat type.

Supplementary Table 3. List of full-text articles or species excluded from our meta-analysis and the reasons for exclusion

Study	Taxa	Species excluded	Reason for exclusion
Full-text articles excluded			
Lunardi & Macedo[3]	Bird	*Charadrius semipalmatus* and *Calidris pusilla*	Species-specific data not provided
Møller[4]	Bird	71 species	Insufficient species-specific data to calculate effect size
Møller *et al.*[5]	Bird	39 species	Species-specific data not provided
Lehrer *et al.*[6]	Mammal	*Marmota monax*	Only model selection results were presented without statistical results to calculate effect sizes
Smith *et al.*[7]	Mammal	*Ursus americanus*	FID data were not presented isolated for human approach only
Species information excluded from full-text articles included in our data set			
Møller & Tryjanowski[8]	Bird	*Picus viridis* and *Dendrocopos major*	Of 42 species, these two species had no information about sample size
Carrete & Tella[9]	Bird	*Molothrus badius*, *Myiopsitta monachus*, *Polyborus plancus*, and *Sicalis flaveola*	Of 26 species, these species had no information on variance of flight initiation distance of the urban population
Díaz *et al.*[10]	Bird	*Carduelis flammea*, *Cygnus olor*, *Haematopus ostralegus*, *Phasianus colchicus*, and *Rissa tridactyla*	Of 83 species with complete information to calculate effect sizes, we used only those species in which urban and rural population were from the same country (78 species)

Supplementary Table 4. Model selection for the most parsimonious combination among three random factors

model	phylogeny	species	study	AICc	ΔAICc
all taxa					
model 1				1453.20	111.73
model 2	●			1431.99	90.52
model 3		●		1445.30	103.82
model 4			●	1365.71	24.23
model 5	●	●		1432.59	91.11
model 6*	●		●	1341.47	0
model 7		●	●	1358.13	16.65
model 8	●	●	●	1343.46	1.98
all birds					
model 1				1286.93	87.21
model 2	●			1265.85	66.13
model 3		●		1276.29	76.57
model 4			●	1223.43	23.70
model 5	●	●		1267.88	68.16
model 6*	●		●	1199.72	0
model 7		●	●	1215.19	15.47
model 8	●	●	●	1201.77	2.04
urban birds					
model 1				870.73	39.14
model 2	●			852.55	20.97
model 3		●		865.26	33.68
model 4			●	855.14	23.56
model 5	●	●		854.60	23.02
model 6*	●		●	831.58	0
model 7		●	●	847.83	16.25
model 8	●	●	●	833.64	2.06

The random factors are the phylogenetic relatedness of species to account for phylogenetic inertia (phylogeny), the species identity to account for multiple effect sizes per species (species), and the study identity to account for multiple effect sizes per study (study). AICc, Akaike information criteria corrected by sample size. ΔAICc, difference in relation to the best model, i.e. the model with the lowest AICc. The best models are indicated by asterisks.

Supplementary Table 5. Correlation matrix (Pearson's *r*) of the covariates explored in the model selections

	**Body mass**	**Clutch size**	**Diet**	**Foraging habit**	**Group size**	**Habitat openness**	**Migration**
**Body mass**	-	-0.23	0.21	0.42	0.29	-0.42	-0.05
**Clutch size**	-0.26	-	0.17	-0.27	0.02	0.24	-0.16
**Diet**	0.09	0.17	-	0.08	0.16	0.05	-0.02
**Foraging habit**	0.33	-0.23	-0.04	-	0.29	-0.10	-0.10
**Group size**	0.23	0.004	0.09	0.28	-	-0.10	-0.10
**Habitat openness**	-0.40	0.32	0.13	-0.38	-0.09	-	0.03
**Migration**	-0.02	-0.13	-0.02	-0.04	-0.12	-0.02	-

Values within grey cells are from covariates used in model selection using all bird species, whereas values within white cells are from covariates used in model selection using only birds from rural-urban habitat contrasts. The highest correlations found were between body mass and foraging habit (r = 0.42) and between body mass and habitat openness (r = –0.42).

Supplementary Methods

Phylogeny reconstruction

To obtain the phylogeny of birds species for our dataset, we used the time-calibrated avian phylogeny[11] available at http://birdtree.org/. Time-calibrated phylogenies were also available for mammals[12] and lizards[13]. We used the function *prune.sample* of the R package Picante[14] v. 1.6-2 to prune the tree so that it only included species present in our data sets.

We used a combined phylogeny of birds, mammals and lizards (Supplementary Fig. 9) to test for differences in the overall effect sizes of these taxa while accounting for their shared evolutionary history. Because we do not have good estimates of basal branch lengths, we only used the topology of the overall tree created by joining all the three phylogenies together. For phylogenetic meta-analysis, we ultrametricized the tree using Grafen's method[15] with *rho* = 1, implemented in the function *compute.brlen* from the R package APE[16] v. 3.1-4. This kind of transformation assumes the Brownian motion model of evolution. Although the use of only one phylogeny in a statistical model does not account for the uncertainty of the tree, the robustness of regression estimates from analysis has been reported[17–19]. To test for the robustness of our findings, we conducted multilevel mixed-effect meta-analysis (using study identity and phylogeny as random factors) for each taxa separately using their time-calibrated trees. These estimates were very similar to those using the combined phylogeny (mean [95% CI]: birds = -1.08 [-1.51, -0.65]; mammals = -1.26 [-1.94, -0.58]; lizards = -1.10[-1.68, -0.52]). Also, it is noted that when phylogenetic signal is weak like in our case, phylogenetic analysis effectively reduces to non-phylogenetic analysis. This is because each data point (i.e. effect sizes) can be considered to be independent of phylogenetic relatedness.

Supplementary References

1. Hedges, L. V. Distribution theory for Glass' s estimator of effect size and related estimators. *J. Educ. Stat.* **6,** 107–128 (1981).

2. Stankowich, T. & Blumstein, D. T. Fear in animals: a meta-analysis and review of risk assessment. *Proc. R. Soc. B* **272,** 2627–2634 (2005).

3. Lunardi, V. O. & Macedo, R. H. Shorebirds can adopt foraging strategies that take advantage of human fishing practices. *Emu* **114,** 50–60 (2014).

4. Møller, A. P. Urban areas as refuges from predators and flight distance of prey. *Behav. Ecol.* **23,** 1030–1035 (2012).

5. Møller, A. P., Grim, T., Ibáñez-Álamo, J. D., Markó, G. & Tryjanowski, P. Change in flight initiation distance between urban and rural habitats following a cold winter. *Behav. Ecol.* **24,** 1211–1217 (2013).

6. Lehrer, E., Schooley, R. & Whittington, J. Survival and antipredator behavior of woodchucks (*Marmota monax*) along an urban–agricultural gradient. *Can. J. Zool.* **90,** 12–21 (2012).

7. Smith, T. S., Oyster, J., Partridge, S. D., Martin, I. E. & Sisson, A. Assessing American black bear response to human activity at Kenai Fjords National Park, Alaska. *Ursus* **23,** 179–191 (2012).

8. Møller, A. P. & Tryjanowski, P. Direction of approach by predators and flight initiation distance of urban and rural populations of birds. *Behav. Ecol.* **25,** 960–966 (2014).

9. Carrete, M. & Tella, J. L. Inter-individual variability in fear of humans and relative brain size of the species are related to contemporary urban invasion in birds. *PLoS One* **6,** e18859 (2011).

10. Díaz, M. *et al.* The geography of fear: a latitudinal gradient in anti-predator escape distances of birds across Europe. *PLoS One* **8,** e64634 (2013).

11. Jetz, W., Thomas, G. H., Joy, J. B., Hartmann, K. & Mooers, A. O. The global diversity of birds in space and time. *Nature* **491,** 444–448 (2012).

12. Bininda-Emonds, O. R. P. *et al.* The delayed rise of present-day mammals. *Nature* **446,** 507–512 (2007).

13. Pyron, R. A. & Burbrink, F. T. Early origin of viviparity and multiple reversions to oviparity in squamate reptiles. *Ecol. Lett.* **17,** 13–21 (2014).

14. Kembel, S. W. *et al.* Picante: R tools for integrating phylogenies and ecology. *Bioinformatics* **26,** 1463–1464 (2010).

15. Grafen, A. The phylogenetic regression. *Philos. Trans. R. Soc. London B* **326,** 119–157 (1989).

16. Paradis, E., Claude, J. & Strimmer, K. APE: Analyses of Phylogenetics and Evolution in R language. *Bioinformatics* **20,** 289–290 (2004).

17. Rohlf, F. J. A comment on phylogenetic correction. *Evolution* **60,** 1509–1515 (2006).

18. Hadfield, J. D., Krasnov, B. R., Poulin, R. & Nakagawa, S. A tale of two phylogenies: comparative analyses of ecological interactions. *Am. Nat.* **183,** 174–87 (2014).

19. Stone, E. A. Why the phylogenetic regression appears robust to tree misspecification. *Syst. Biol.* **60,** 245–260 (2011).
